# Co-infection of Chicken Layers With *Histomonas meleagridis* and Avian Pathogenic *Escherichia coli* Is Associated With Dysbiosis, Cecal Colonization and Translocation of the Bacteria From the Gut Lumen

**DOI:** 10.3389/fmicb.2020.586437

**Published:** 2020-10-30

**Authors:** Mohamed Kamal Abdelhamid, Narciso M. Quijada, Monika Dzieciol, Tamas Hatfaludi, Ivana Bilic, Evelyne Selberherr, Dieter Liebhart, Claudia Hess, Michael Hess, Surya Paudel

**Affiliations:** ^1^Clinic for Poultry and Fish Medicine, Department for Farm Animals and Veterinary Public Health, University of Veterinary Medicine Vienna, Vienna, Austria; ^2^Department of Pathology, Faculty of Veterinary Medicine, Beni-Suef University, Beni-Suef, Egypt; ^3^Department for Farm Animals and Veterinary Public Health, Institute of Food Safety, Food Technology and Veterinary Public Health, University of Veterinary Medicine Vienna, Vienna, Austria; ^4^Division of Microbial Ecology, Department of Microbiology and Ecosystem Science, University of Vienna, Vienna, Austria; ^5^Christian Doppler Laboratory for Innovative Poultry Vaccines (IPOV), University of Veterinary Medicine Vienna, Vienna, Austria

**Keywords:** *Histomonas meleagridis*, *Escherichia coli*, histomonosis, colibacillosis, 16S rRNA gene amplicon sequencing, microbiota, immunohistochemistry, translocation

## Abstract

Histomonosis in chickens often appears together with colibacillosis in the field. Thus, we have experimentally investigated consequences of the co-infection of birds with *Histomonas meleagridis* and avian pathogenic *Escherichia coli* (APEC) on the pathology, host microbiota and bacterial translocation from the gut. Commercial chicken layers were infected via oral and cloacal routes with *lux*-tagged APEC with or without *H. meleagridis* whereas negative controls were left uninfected. Except one bird, which died due to colibacillosis, no clinical signs were recorded in birds infected with bioluminescence *lux* gene tagged *E. coli.* In co-infected birds, depression and ruffled feathers were observed in 4 birds and average body weight gain significantly decreased. Typhlitis caused by *H. meleagridis* was present only in co-infected birds, which also had pronounced microscopic lesions in systemic organs such as liver, heart and spleen. The 16S rRNA gene amplicon sequencing showed that in co-infected birds, corresponding to the severity of cecal lesions, microbial species richness and diversity in caeca greatly decreased and the abundance of the *Escherichia* group, *Helicobacter* and *Bacteroides* was relatively higher with a reduction of commensals. Most of the shared Amplicon Sequencing Variants between cecum and blood in co-infected birds belonged to *Pseudomonas, Staphylococcus, and* members of *Enterobacteriaceae* while those assigned as *Lactobacillus* and members of *Ruminococcaceae* and *Lachnospiraceae* were found mainly in negative controls. In infected birds, *E. coli* in the cecal lumen penetrated into deeper layers, a phenomenon noticed with higher incidence in the dead and co-infected birds. Furthermore, numbers of *lux*-tagged *E. coli* in caeca were significantly higher at every sampling date in co-infected birds. Altogether, infection of layers with *H. meleagridis* and *E. coli* resulted in more severe pathological changes, dramatic shift in the cecal mucosa-associated microbiota, higher tissue colonization of pathogenic bacteria such as avian pathogenic *E. coli* in the gut and increased penetration of *E. coli* from the cecal lumen toward peritoneum. This study provides novel insights into the parasite-bacteria interaction *in vivo* highlighting the role of *H. meleagridis* to support *E. coli* in the pathogenesis of colibacillosis in chickens.

## Introduction

Histomonosis caused by the flagellated protozoan parasite *Histomonas meleagridis* is of growing attentions in the poultry industry especially, due to the lack of licensed drug for therapy and prophylaxis (Clark and Kimminau, 2017; Liebhart et al., 2017). Turkeys elicit less effective host immune responses against the parasite and thus they are more prone to the disease as compared to chickens (Mitra et al., 2018). Nevertheless, experimental infection of layers with *H. meleagridis* resulted in severe inflammation of caeca and drop in egg production (Liebhart et al., 2013). In addition, previously reported field cases in layers and breeders as well as a high seroprevalence of the parasite in commercial layer flocks underline the significance of *H. meleagridis* in chickens (Grafl et al., 2011; van der Heijden and Landman, 2011; Hess et al., 2015). Beside its role as a primary pathogen, *H. meleagridis* might also act as a predisposing factor for inducing other diseases such as colibacillosis, a disease caused by avian pathogenic *Escherichia coli* (APEC), which is more likely to appear in birds with a compromised mucosal barrier (Nolan et al., 2020). In adult chickens, colibacillosis is characterized by lesions such as aerosacculitis, perihepatitis, pericarditis, egg peritonitis and salpingitis (Lister and Barrow, 2008). *In vitro* characterization of *E. coli* isolates obtained from clinical cases of histomonosis together with colibacillosis in chickens indicated a dissemination of intestinal *E. coli* isolates to systemic organs (Paudel et al., 2018), however, the mechanism is not yet understood.

A normal gut microbiota is necessary for maintaining the intestinal homeostasis, nutrient digestion, immune response regulation and a protective barrier through competitive exclusion and pathogen colonization inhibition (Yegani and Korver, 2008). Any qualitative or quantitative changes in the normal microbial community is called dysbiosis, which leads to a decrease of intestinal wall barrier function and increased risk for bacterial translocation (Berg, 1999; Teirlynck et al., 2011). In order to identify intestinal microbial communities in the host, traditional culture-based methods are less attractive due to the fact that large proportion of bacteria do not grow *in vitro* or require highly selective growth conditions which might leave them unrecognized (Hugenholtz et al., 1998). In this regards, the application of high throughput 16S rRNA gene amplicon sequencing technology has rapidly accelerated for the determination of bacterial community census in the gastrointestinal tract (Shang et al., 2018). The same technique was also applied to get insights on richness and diversity of blood microbial communities in humans and animals (Païssé et al., 2016; Vientos-Plotts et al., 2017). In chickens, characterization of blood microbiota is still not common and was previously reported only in a single study in broilers in context with bacterial chondronecrosis and osteomyelitis (Mandal et al., 2016).

The outstanding interplay between *H. meleagridis* and bacteria has been largely elucidated with *in vitro* studies but the parasite-bacteria interaction in the host is still not fully understood (Bilic and Hess, 2020). Thus, the present study was conducted in laying hens with the following four objectives: (a) to examine the pathological severity due to *E. coli* with or without *H. meleagridis* (b) to assess the influence of *E. coli* with or without *H. meleagridis* on cecal and blood microbial population (c) to explore the cecal invasion of *E. coli* in birds with or without histomonosis and finally, (d) to investigate the influence of *H. meleagridis* on the bacterial load and systemic translocation by tracing a specific *lux*-tagged pathogenic strain of *E. coli*.

## Materials and Methods

### Birds and Housing

The animal trial was approved by the institutional ethics committee and the national authority according to §8ff of the law for Animal Experiments, Tierversuchsgesetz-TVG (License Number GZ.68.205/0220-V/3b/2018).

Forty-eight 20-week-old commercial layer pullets (Schropper GmbH, Gloggnitz, Austria) were divided into three groups with 16 birds each. Birds were subcutaneously tagged with an individual numbered tag (Swift tag) and each group was housed separately in a negatively pressured room on deep litter. Feed and water were provided *ad libitum*, except for 3 h after each infection.

### Preparation of *H. meleagridis* and *E. coli* for Infection

#### H. meleagridis

The virulent monoxenic clonal culture *H. meleagridis/*turkey/Austria/2922-C6/04 (Hess et al., 2006) co-cultivated with the laboratory strain *E. coli* DH5α (Invitrogen, Vienna, Austria) (Ganas et al., 2012) was *in vitro* passaged 24 times prior to infection. The inoculum consisted of *H. meleagridis* cells in Medium 199 with Earle’s salts, L-glutamine, 25 mM HEPES and L-amino acids (Gibco^TM^ Invitrogen, Austria), 15% fetal calf serum (Gibco^TM^ Invitrogen) and 0.25% (w/v) rice starch (Sigma-Aldrich, Vienna, Austria).

#### E. coli

The strain PA14/17480/5-ovary was isolated from a diseased bird and represents the pathotype called avian pathogenic *E. coli* (APEC) (Rezaee et al., 2020). Site specific chromosomal insertion of *lux*ABCDE operon was performed in this strain for constitutive expression of bioluminescence following a previously published protocol (Riedel et al., 2007). In order to confirm the integration of the *lux* cassette, we sequenced the whole genome of the parent and *lux*-tagged strains (data not shown). Based on this information, the following two pairs of primers were designed targeting both flanking regions of *luxA*BCDE insertion site and the integration of the operon was validated with a conventional PCR: set1-F 5′ GGGTAGAATTCCAGGTGTAGC 3′, set1-R 5′ GGATTCAGGAGTGGACAGAAC 3′ and set2-F 5′ CGTGCCCATATTCTTGAGC 3′, set2-R 5′ GCCCCCGTCAATTCATTTG 3′. For inoculum preparation, one loopful of *lux*-tagged *E. coli* stock culture was plated onto Luria Bertani (LB) agar plates with erythromycin (+ery) and incubated at 37°C for 24 h. Selected fresh colonies were inoculated overnight in LB + ery broth at 37°C with agitation. On the day of infection, bacterial pellets were washed twice and suspended in phosphate-buffered saline (PBS). Bacterial concentrations were determined with spectrophotometer (OD600) and by colony forming unit (CFU/ml) counts.

### Experimental Design

At 23 weeks of age, birds in group 1 were inoculated with 0.6 ml of 1 × 10^6^ cells/ml of *H. meleagridis* equally divided orally and cloacally as described previously (Liebhart et al., 2013). Likewise, birds in groups 2 and 3 received the same volume of culture medium via the two routes. Subsequently, at 2, 4, and 6 days post *H. meleagridis* infection (dpi), 0.5 ml of 5.3–6.7 × 10^8^ CFU/ml of *lux*-tagged *E. coli* suspensions were inoculated in birds in groups 1 and 2 via oral and cloacal routes. At these time points, negative control birds in group 3, received 0.5 ml of sterile PBS via the same two routes. All birds were observed daily for clinical signs. Necropsy and sampling were performed in four birds from each group at 7, 10, 14, and 28 dpi with an exception at 7 dpi when only three birds from group 2 were killed.

### Body Weight and Necropsy

Body weight of euthanized birds as well as gross pathological lesions in the cecum and systemic organs associated with *H. meleagridis* and/or *E. coli* infections were recorded during necropsy according to a standard protocol. Severity of cecal lesions were categorized based on a previously established lesion scoring scheme ranging from lesion score (LS) 0 to 4 (Windisch and Hess, 2010).

### Histopathology

**FIGURE 1:**
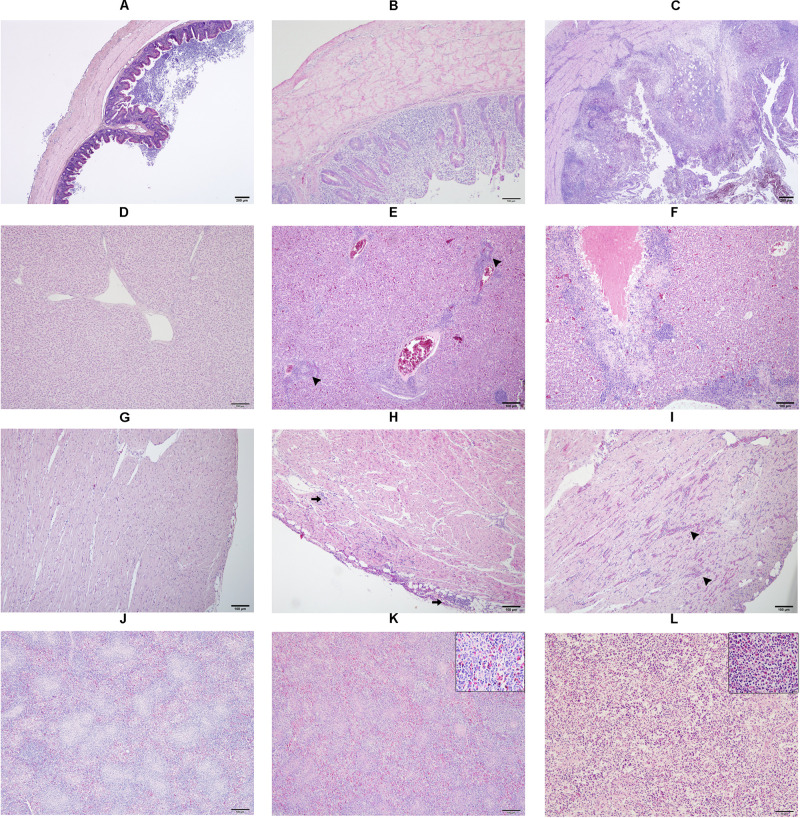
Microscopic lesion scores (LS) in caecum **(A–C)**, liver **(D–F)**, heart **(G–I)** and spleen **(J–L)** of commercial chicken layers infected with a *lux*-tagged *E. coli* with or without *H. meleagridis.* Caeca: normal without pathological changes, LS0 **(A)**; epithelial erosion with infiltration of inflammatory cells in the mucosa, LS1 **(B)**; severe fibrino-necrotic typhlitis with transmural inflammatory cell infiltrate, LS2 **(C)**. Liver: normal without pathological changes, LS0 **(D)**; congestion with periportal infiltration of inflammatory cells (arrow heads), LS1 **(E)**; coalescing necrotizing hepatitis surrounded mostly by lymphocytic cells, LS2 **(F)**. Heart: normal without pathological changes, LS0 **(G)**; cellular infiltrate in the epicardium that extended to the myocardium (arrows), LS1 **(H)**; multifocal interstitial inflammatory infiltrates, mostly granulocytes as indicated by arrow heads, LS2 **(I)**. Spleen: normal without pathological changes LS 0 **(J)**; mild heterophilic infiltration (inset), LS1 **(K)**; severe heterophilic infiltration with lymphoid depletion (inset), LS 2 **(L)**.

Samples from cecum, liver, heart, spleen, ovary and oviduct were collected in 10% neutral buffered formalin during necropsy. Later, tissues were processed for paraffin embedding, sectioned into 5 μm slices and stained with hematoxylin and eosin following a routine protocol before microscopic examinations were performed. A detail description of lesion scores in cecum, liver, heart and spleen from 0 to 2 is provided in Supplementary Table 1 and exemplarily shown in Figure 1.

### Blood Collection and Buffy Coat Preparation

Blood samples were collected for 16S rRNA gene amplicon sequencing analysis. The same four birds from each group were sampled at 0, 7, 10, 14, and 28 dpi. In addition, blood from birds killed at 7, 10, and 14 dpi were also taken. Before drawing blood, the area around the wing vein was cleaned and disinfected with 70% alcohol. After that 4 ml of blood was aseptically collected from the wing vein directly into blood collection tubes containing K2EDTA anticoagulant (VACUETTE^®^ Tube, Greiner Bio-One GmbH, Austria) and mixed gently. Buffy coat preparation was done for microbiome analysis as described previously (Mandal et al., 2016). For this, blood samples were centrifuged at 5000× rpm for 5 min at room temperature and buffy coat layers from each tube were collected under a laminar hood with a micropipette. The samples were stored at −20°C until further use. In a preliminary study, suitability of the buffy coat over whole blood was determined in our conditions by quantifying 16S rRNA gene of total bacterial communities in whole blood or buffy coat samples. Different combinations of centrifugation speed and time were tested after which results confirmed that DNA yields were higher in buffy coats prepared after centrifugation of blood samples at 5000x rpm for 5 min (data not shown).

### DNA Extraction and qPCR Amplification

Cecal tissues from birds killed at 7, 10, 14, and 28 dpi were collected for amplicon sequencing. For this, caeca were opened and fecal materials were gently removed without damaging tissues. Mucosal tissues were scrapped with scalpel and collected in Eppendorf tubes. DNA extraction was done from 25 μl of buffy coat samples and 25 mg of cecal samples using DNeasy Blood and Tissue Kit (QIAGEN, Hilden, Germany) following manufacturer’s guidelines. DNA concentration was determined using the Qubit^®^ dsDNA BR Assay Kit (Thermo Fisher Scientific, Vienna, Austria) and Qubit^®^ 2.0 Fluorometer (Thermo Fisher Scientific) according to the manufacturer’s instructions.

The 16S rRNA gene PCR quantification of the total bacterial cell equivalents (BCE) in blood and cecal samples was done based on a published protocol (Metzler-Zebeli et al., 2013). Standard curves were constructed from serially diluted PCR products by using the primer set 341F (5′-CCTACGGGAGGCAGCAG-3′ and 534R (5′-ATTACCGCGGCTGCTGG-3′) (Muyzer et al., 1993). Extraction controls (*n* = 14) as well as non-template controls (NTCs) were included in each qPCR run in order to identify possible contamination. The final copy numbers of total bacteria were calculated using the quantitative mean of the bacterial cell equivalents (BCE) per 25 mg of cecal tissues and 25 μl of buffy coat. Additionally, an average of four 16S rRNA gene copies per genome was taken into account when extrapolating of extracted DNA (100 μl) (Větrovský and Baldrian, 2013).

### Illumina Amplicon Sequencing

For Illumina MiSeq Amplicon sequencing, 95 buffy coat, 48 cecum and 14 extraction control samples were processed. The variable region V3/V4 of the 16S rRNA gene was targeted using the published gene-specific sequences (Klindworth et al., 2013). Illumina adapter overhang nucleotide sequences were added to gene-specific sequences. The resulting full-length primer sequences used were 16S forward primer 5′TCGTCGGCAGCGTCAGATGTGTATAAGAGACAGCCTAC GGGNGGCWGCAG, reverse primer 5′ GTCTCGTGGGCT CGGAGATGTGTATAAGAGACAGGACTACHVGGGTATCTA ATCC. Library preparation including sample quantity control, NextEra two-step PCR amplification, equimolar pooling of samples and sequencing with a 250 bp paired-end read protocol (V3-V4) using an Illumina MiSeq sequencing platform were performed by the Next Generation Sequencing facility of the Vienna BioCenter Core Facilities,^1^ Austria.

### Bioinformatics and Data Analysis

Raw demultiplexed sequencing data quality was inspected by using FASTQC^2^ and remaining primers and barcodes were removed by using Trimmomatic (Bolger et al., 2014). The resulting sequencing data were processed by using QIIME2 version 2019.7 (Bolyen et al., 2019) pipeline. *q2*-*dada2* (Callahan et al., 2016) and *q2-feature-table*^3^ plugins were used for quality filtering of the reads, merging of the paired ends, removal of chimeras and resolution of Amplicon Sequence Variants (ASVs). ASVs rely on single nucleotide differences between sequences and can be considered as Operational Taxonomic Units (OTUs) clustered at 100% identity threshold. The resulting ASV table was then imported to the R environment (v3.6.1)^4^ and the *decontam* package (Davis et al., 2018) was used to identify and remove those ASVs considered as potential contaminants according to the information contained in the extraction controls dataset by following the recommendations form the developers. *Decontam* analysis was performed independently over cecum and blood data, as they represent different environments with different biomass, and yielded differences in extracted DNA concentration. Additionally, extraction controls were divided between the two performed sequencing runs. The *decontam* was also performed regarding this information, in order to consider contaminants from the sequencing machines. For cecum sequencing data, the argument *IsContam* was parsed to *decontam* and 182 ASVs (that represented 2.4% of all 16S rRNA gene amplicon sequences from chicken cecum) were identified as potential contaminants and removed. Blood is considered to contain lower biomass so the argument *IsNotContam* was parsed to *decontam*, as recommended by the *decontam* developers. A total of 2,263 ASVs were identified as contaminants and removed from the blood dataset, which resulted in a total loss of 83.8% of all 16S rRNA gene amplicon sequences from chicken blood.

The resulting ASV tables were converted to BIOM format (McDonald et al., 2012) and imported to QIIME2. Alpha rarefaction curves and Good’s coverage were calculated by using the *q2-diversity*^5^ plugins, revealing that the microbial diversity after quality control and contamination removal was sufficiently covered. A phylogenetic tree was built using *q2-alignment* (Katoh and Standley, 2013) and *q2-phylogeny* (Price et al., 2010) plugins. A pre-trained Naïve Bayes classifier based on SILVA database (Pruesse et al., 2007), previously trimmed to harbor the V3-V4 region of 16S rRNA gene (by following the instructions from https://docs.qiime2.org/2019.7/tutorials/feature-classifier/), was used for taxonomy assignment of the identified ASVs by using the *q2-feature-classifier* plugin (Bokulich et al., 2018). ASVs and their representative sequences that got a taxonomic assignation to “Chloroplast”, “Eukaryota,” or “Mitochondria” were removed from the dataset by using the *filter-table* option of the *q2-taxa* plugin. Alpha-and beta-diversity were analyzed by using *q2-diversity* (see text footnote 5) and *q2-taxa*^6^ plugins. Chao1 (Chao, 1984), Shannon (Shannon, 1948) and Simpson (Simpson, 1949) metrics, for the calculation of species richness, diversity and evenness, respectively, were calculated by using *phyloseq* (McMurdie and Holmes, 2013). For beta-diversity studies, cecum and blood samples’ ASV tables were rarefied to 80,451 and 5,167 sequences per sample, respectively, in order to avoid biases due to different sequencing depths. Bray-Curtis dissimilarity (Bray and Curtis, 1957) distance matrices were calculated. Plotting was carried out in R environment by using *dplyr*,^7^ *ggplot2* (Wickham, 2016), *lemon*^8^, *plyloseq* (McMurdie and Holmes, 2013) and *rehsape2* (Wickham, 2007) packages.

### Immunohistochemistry

Formalin fixed and paraffin embedded samples from cecum, liver, heart and spleen of four birds of each group at 14 dpi were processed for immunohistochemistry (IHC) for the detection of *H. meleagridis* following a published protocol (Singh et al., 2008). The same protocol was used for the localization of *E. coli* in the mentioned organs from all the birds. Briefly, tissue sections mounted on glass slides were dewaxed in xylene, rehydrated and subjected to antigen retrieval by heating in 0.01M citric buffer (pH 6.0) for 10 min. Following treatment with 1.5% hydrogen peroxide for blocking endogenous peroxidase activity, non-specific background staining was blocked by incubating the samples with goat serum for 30 min. Tissues were incubated overnight at 4°C with the primary antibody (rabbit anti-*H. meleagridis* antibody in a dilution of 1: 10000, respectively, anti-*E. coli* LPS antibody (2D7/1), ab35654, Abcam, Austria in a dilution of 1:500). On the next day, biotinylated anti-rabbit, respectively, anti-mouse IgG antibody (Vector Laboratories, Austria) was added on the samples and incubated for 1 h at room temperature. The Vectastain ABC Kit and DAB Peroxidase Substrate Kit (Vector Laboratories) were used for visualizing the pathogens, which stained brown in contrast to the surrounding tissue. Mayer’s hematoxylin (Merck KGaA, Austria) was used for counterstaining.

### Bacteriology

Bacteriological examination was performed to re-isolate and quantify the experimentally inoculated *lux*-tagged strain of *E. coli* in different organs. For direct plating, heart, lung and ovary from all birds necropsied at 4, 7, 10, 14, and 28 dpi were streaked directly on LB + ery agar plates. Similarly, at 7, 10, 14, and 28 dpi, 500 μl of the whole blood from all birds were plated on agar plates. For bacterial quantification, tissue samples from cecum, liver and spleen were homogenized and 10-fold diluted suspensions were plated in duplicates. Following incubation at 37°C for 24 h, agar plates were visualized under the *In Vivo* Imaging System (IVIS) instrument with binning of 16 (large) and an f/stop of 1 (IVIS Lumina LT, PerkinElmer, Rodgau, Germany) and bioluminescent bacterial colonies were counted (Supplementary Figure 1). Based on the colony forming unit counts, bacterial load of *lux*-tagged *E. coli* in organs were expressed as CFU/g.

### Statistical Analysis

Data on body weight were analyzed with R (R Core Team, 2017, R Foundation for Statistical Computing, Vienna, Austria; see text footnote 4). ANCOVA model was applied with group as factor, dpi as covariate. Data on mean histopathological lesion scores, mean log of 16S rRNA gene copy numbers and mean log CFU/g in different organs were analyzed with one-way ANOVA and Tukey’s multiple comparison was done in the *post hoc* test. The Spearman’s rank correlation coefficient (r) was computed to evaluate the correlation between macroscopic cecal lesion score with body weight, microbial species diversity indices and bacterial load of *lux*-tagged *E. coli* in the cecum. Data on alpha diversity indices from cecal samples were analyzed using the Kruskal–Wallis test for overall significance followed by Dunn’s multiple comparisons test for pairwise comparison between groups. All these tests were performed in SPSS (IBM^®^ SPSS^®^ version 25; IBM Cooperation, New York, United States). PERMANOVA (Permutational multivariate analysis of variance) test was performed over the Bray-Curtis distance matrices at each time point by using the q2-beta-group-significance (Anderson, 2005) plugin of QIIME2 (v2019.7) to evaluate differences in beta diversity between groups. In all cases, *p* ≤ 0.05 was considered as statistically significant.

## Results

### Clinical Signs

In group 1, clinical signs characterized by depression, ruffled feathers and drowsiness were observed in 1, 2, and 2 birds at 8, 12, and 16 dpi, respectively. In group 2, one bird showed acute progression of clinical signs with severe depression, loss of appetite, closed eyes, reluctance to move at 4 dpi, and died shortly after the onset of clinical symptoms. No clinical signs were observed in negative control birds from group 3 until the end of the study.

### Gross Lesions and Body Weight

**FIGURE 2:**
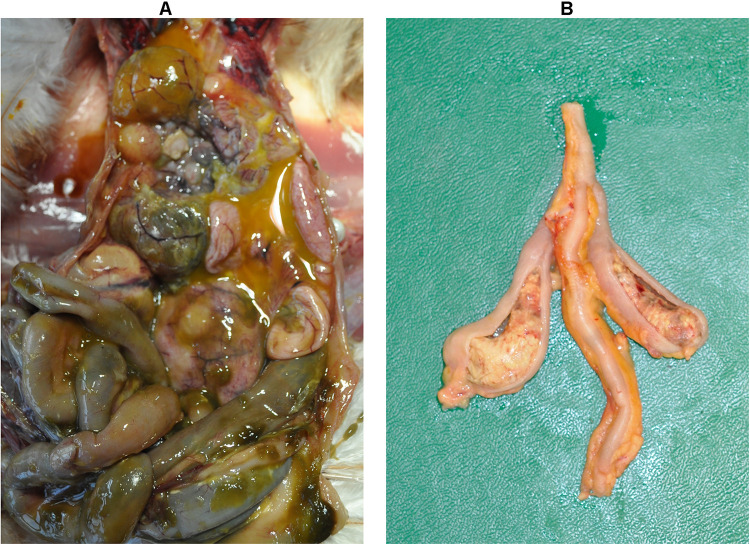
Macroscopic lesions. Severe peritonitis in the dead bird due to infection with a *lux*-tagged avian pathogenic *E. coli* **(A)**; thickening of the cecal wall with fibrinous mass in the lumen caused by *H. meleagridis* infection (LS4, group 1) **(B)**.

The dead bird from group 2 showed severe peritonitis (Figure 2A) accompanied with aerosacculitis, pericarditis, perihepatitis, and salpingitis. Pathomorphological changes in caeca were exclusively observed in birds from group 1. A representative cecum with LS4 is shown in Figure 2B. At 7 dpi, two birds from group 1 had LS3 in caeca and one bird showed degeneration of follicles and oviduct (Table 1). At this time point, two birds from group 2 had mild cloudy air sacs. At 10 dpi, LS3 and LS4 were recorded in caeca of two birds from group 1, whereas one bird from group 2 had degenerated follicles and oviduct. Subsequently, at 14 dpi, two birds from group 1 showed LS4 and one bird had LS2 in caeca. In addition, mild cloudy air sacs in 3 birds and degenerated follicles and oviduct in one bird were recorded from the same group. At 28 dpi, cecal LS3 and LS4 were observed in two birds from group 1. Other findings comprised atrophy of ovary and oviduct in one bird and mild cloudy air sac in two birds from the same group.

**TABLE 1:** Macroscopic lesions in caeca and systemic organs.

dpi^a^	Group (number of birds sampled)^b^	Macroscopic lesions in infected birds			
		Mean cecal lesion score (mean ± SEM)	No. of birds that showed cecal lesions	Lesions in systemic organs	No. of birds that showed lesions in systemic organs
4	2 (*n* = 1)	–^c^	–	Cloudy air sac, perihepatitis, salpingoperitonitis and congested ovary	1
7	1 (*n* = 4)	1.5 ± 0.87	2	Degenerated follicles and oviduct	1
	2 (*n* = 3)	–	–	Cloudy air sacs	2
10	1 (*n* = 4)	1.75 ± 1	2	–	–
	2 (*n* = 4)	–	–	Degenerated follicles and oviduct	1
14	1 (*n* = 4)	2.5 ± 0.96	3	Cloudy air sacs Degenerated follicles and oviduct	3 1
	2 (*n* = 4)	–	–	–	–
28	1 (*n* = 4)	1.75 ± 1	2	Cloudy air sacs Degenerated ovary and oviduct	2 1
	2 (*n* = 4)	–	–	–	–

*^*a*^dpi, days post *H. meleagridis* infection. ^*b*^Group 1, infected with *H. meleagridis* and *lux*-tagged *E. coli*; group 2, infected only with *lux*-tagged *E. coli*. ^*c*^No lesions were observed.*

Birds co-infected with *H. meleagridis* and *lux*-tagged *E. coli* lost weight in comparison to birds from the other two groups, which slightly gained weight until the end of the experiment (Supplementary Figure 2). In pairwise comparisons of body weight taken from euthanized birds from all three groups, the regression slope of the co-infected group was significantly negative, while slopes in groups 2 and 3 were positive but not statistically significant.

### Histopathological Findings

The dead bird from group 2 showed severe inflammation with profuse infiltration of mononuclear cells and intravascular congestion in liver, heart and ovary. Congestion and hemorrgahes within interstitial spaces with lymphoid depletion in spleen and fibrinous serositis of oviduct were also observed.

**FIGURE 3:**
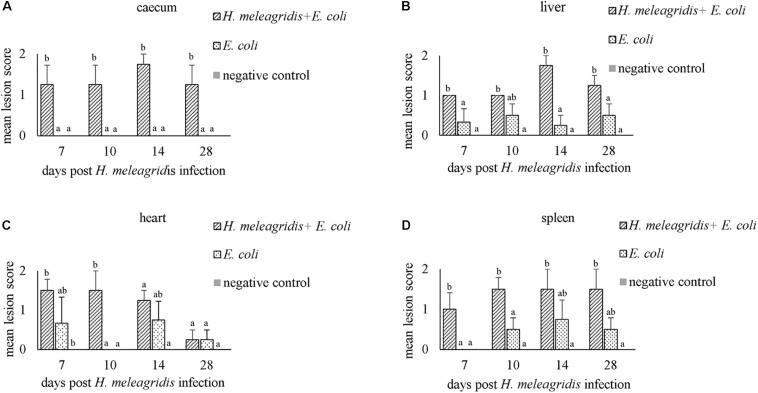
Average histopathological lesion scores in caecum **(A)**, liver **(B)**, heart **(C)** and spleen **(D)**. Bars with different letters within each sampling point denote significantly different values.

In caeca, histopathological lesions were observed only in birds from group 1 and the highest mean LS was recorded at 14 dpi (Figure 3A). Lesions comprised of mucosal erosion with infiltration of inflammatory cells in lamina propria and submucosa (LS1, Figure 1B) or severe destruction of the mucosa and severe inflammation of the entire cecal wall (LS2, Figure 1C).

Mean LSs in the liver were higher in group 1 at every time point and differences with group 2 were statistically significant at 7, 14, and 28 dpi (Figure 3B). In birds from group 2, lesions appeared mild and were not statistically significant to negative controls. Lesions were characterized by infiltration of inflammatory cells and in some cases, accompanied with hepatocellular necrosis (LS1, Figure 1E) or coalescent necrosis with infiltration of inflammatory cells in the liver (LS2, Figure 1F).

In the heart, more birds from group 1 showed lesions but differences in mean LSs with group 2 were statistically significant at 10 dpi (Figure 3C). Lesions were observed also in birds from group 2 at 7, 14 and 28 dpi but were less pronounced. Pathological alterations were mainly characterized with focal myocardial leukocytic infiltration (LS1, Figure 1H) or multifocal cellular infiltrates accompanied by pericarditis (LS2, Figure 1I).

In the spleen, average LSs were significantly higher in group 1 than in group 2 at 7 and 10 dpi (Figure 3D). At 10, 14, and 28 dpi mild lesions were observed also in birds from group 2. Lesions were either mild heterophilic infiltration (LS1, Figure 1K) or severe heterophilic influx within the splenic tissue (LS2, Figure 1L). In some birds from group 1, necrosis and depletion of lymphoid tissues were also seen.

Lymphoid infiltration in the lamina propria and submucosa were seen in oviducts from six birds from group 1. No lesions were observed in the ovary.

### Microbial Composition and Diversity in the Cecum

**FIGURE 4:**
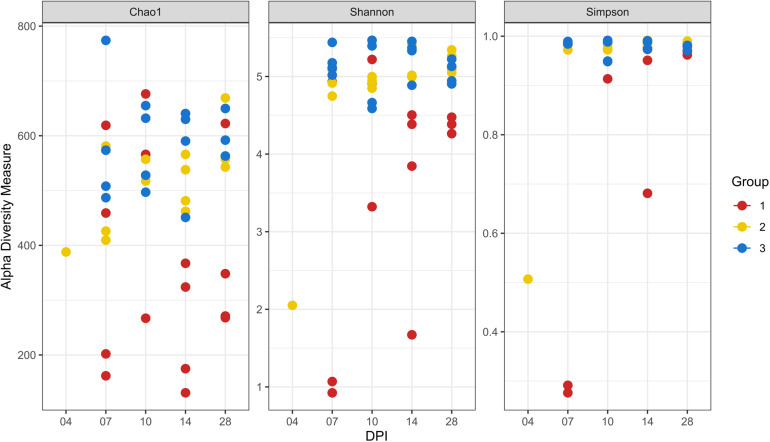
Alpha diversity analyses in cecal samples. Each dot corresponds to one sample. Group 1, infected with *H. meleagridis* + *lux*-tagged *E. coli*; Group 2, infected with *lux*-tagged *E. coli*; Group 3, negative control. dpi denotes for days post *H. meleagridis* infection.

**FIGURE 5:**
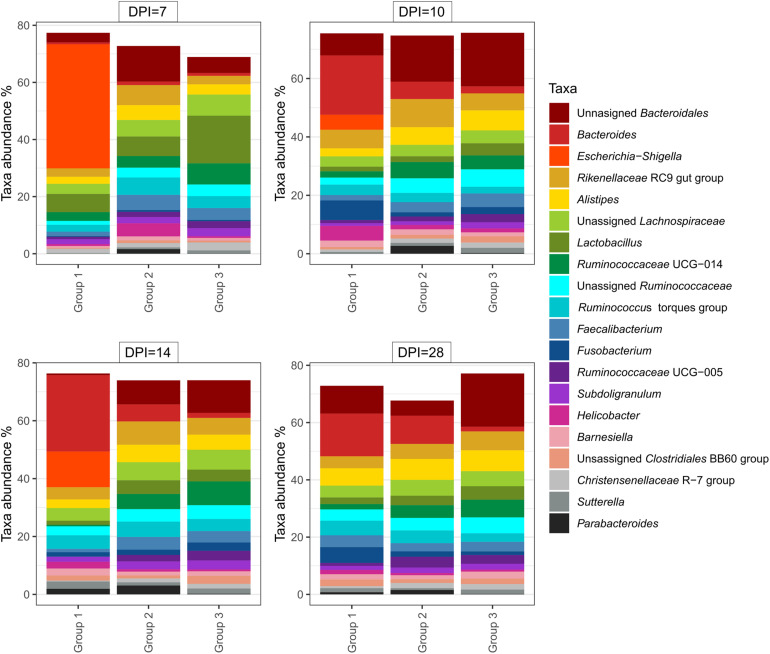
Relative amount (%) of 20 most abundant bacterial genera in caeca of chickens from different groups and days post *H. meleagridis* infection (dpi). Group 1, infected with *H. meleagridis* + *lux*-tagged *E. coli*; Group 2, infected with *lux*-tagged *E. coli*; Group 3, negative control.

At 14 dpi, average total bacterial load in cecum measured by qPCR was significantly lower in group 1 than groups 2 and 3. All other comparisons among groups were not significant (Supplementary Figure 3A). Overall, 14,471 unique ASVs were identified in cecum samples and classified into 18 bacterial and 2 archaeal phyla. *Firmicutes* (48.9%), *Bacteroidetes* (32.5%), *Proteobacteria* (9.4%) and *Fusobacteria* (2.2%) were the most dominant phyla overall and accounted for almost 93% of all the 16S rRNA gene amplicon sequences obtained. Alpha diversity metrics showed that bacterial diversity and richness in co-infected birds (group 1) were substantially decreased, whereas *E. coli* infected (group 2) and negative control (group 3) birds showed similar results (Figure 4). Three samples from group 1 (2 and 1 from 7 and 14 dpi) had low Simpson index values as observed in the dead bird. All these samples were found to be dominated mainly by *Escherichia* ASVs. Upon statistical analysis, the most significant differences in alpha diversity indices between the co-infected and control group were observed at 14 dpi (Supplementary Table 2). Out of 20 most abundant genera, *Escherichia, Bacteroides, Fusobacterium* and *Helicobacter* were higher and *Lactobacillus, Faecalibacterium* and members of the *Ruminococcaceae* family were lower in group 1 as compared to groups 2 and 3 (Figure 5). These patterns were more evident at 7, 10, and 14 dpi.

**FIGURE 6:**
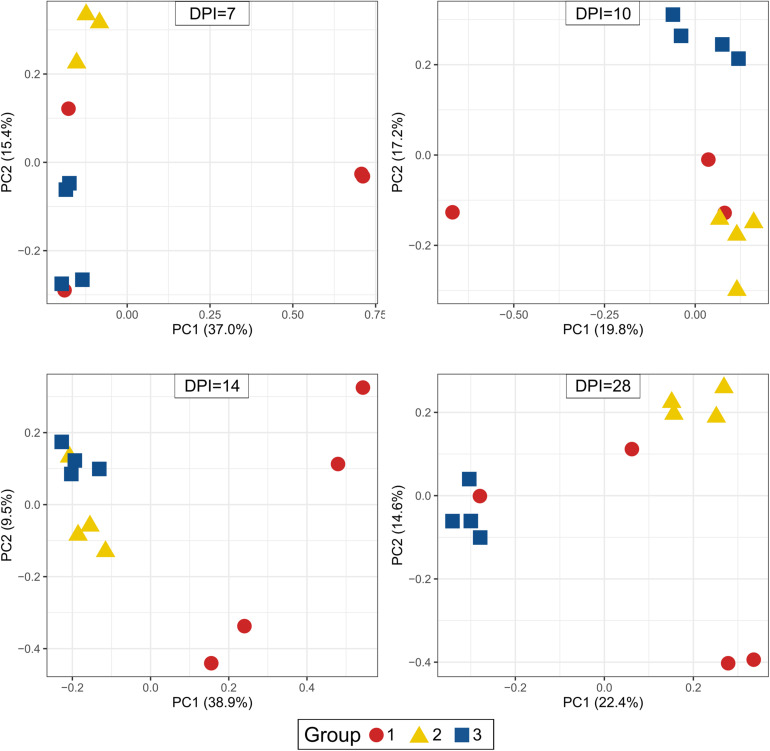
Principal Coordinate Analysis (PCoA) of beta diversity using Bray-Curtis distances. Each dot corresponds to one bird. Group 1, infected with *H. meleagridis* + *lux*-tagged *E. coli*; Group 2, infected with *lux*-tagged *E. coli*; Group 3, negative control. dpi denotes for days post *H. meleagridis* infection.

Beta-diversity of cecum samples was evaluated by using Bray-Curtis distances and shown as Principal Coordinates Analysis (PCoA). Samples from group 1 were very heterogeneous to each other. However, samples from groups 2 and 3 clustered very close to each other within a group at all sampling points post infection, revealing a high homogeneity of bacterial composition within each group throughout the experimental time (Figure 6). Upon statistical analysis for comparison among groups, group 1 differed significantly from groups 2 and 3 at 10, 14, and 28 dpi whereas the differences between groups 2 and 3 were significant at 7, 10, and 28 dpi (Supplementary Table 3).

### Microbial Composition and Diversity in the Blood

Total bacterial load in buffy coat at 10 dpi was significantly higher in birds of groups 1 and 2 as compared to those in group 3 (Supplementary Figure 3B). All other comparisons were not significant. Overall, 16,649 unique ASVs were identified and classified into 25 bacterial and one archaeal phylum. *Firmicutes* (35.1% of all 16S rRNA gene amplicon sequences in blood samples), *Proteobacteria* (28.5%), *Actinobacteria* (15.9%) and *Bacteroidetes* (8.2%) were the most abundant phyla overall and accounted for 87.6% of all 16S rRNA gene amplicon sequences in blood samples. A high proportion of the 16S rRNA gene amplicon sequences (nearly 7.1%, overall), did not get any taxonomic assignment by using the SILVA database.

**FIGURE 7:**
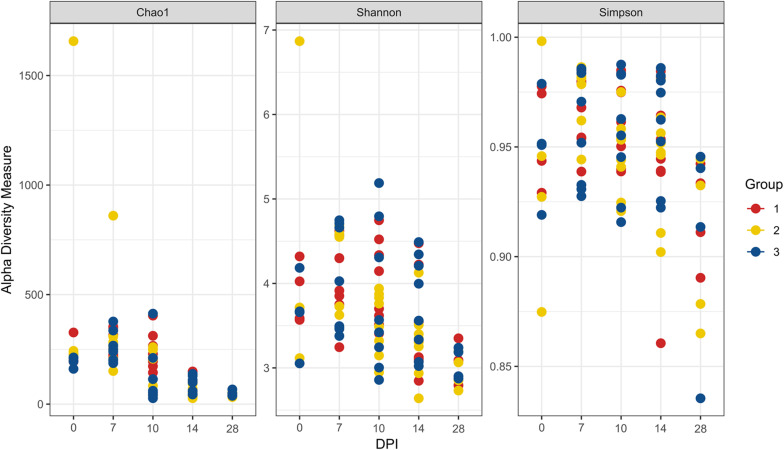
Alpha diversity analyses in blood samples. Each dot corresponds to one bird. Group 1, infected with *H. meleagridis* + *lux*-tagged *E. coli*; Group 2, infected with *lux*-tagged *E. coli*; Group 3, negative control. dpi denotes for days post *H. meleagridis* infection.

**FIGURE 8:**
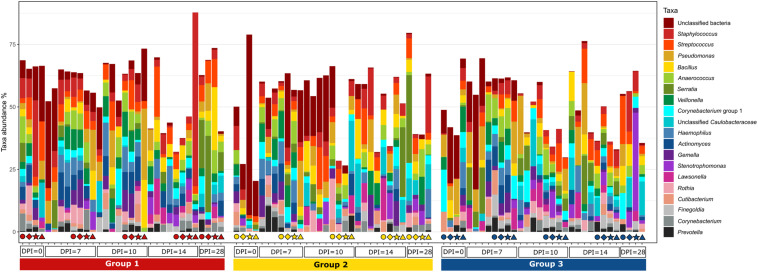
Relative amount (%) of the most abundant bacterial genera present in the blood at different days post *H. meleagridis* infection (0, 7, 10, 14, and 28 dpi). Samples are divided by group. Group 1, infected with *H. meleagridis* + *lux*-tagged *E. coli*; Group 2, infected with *lux*-tagged *E. coli*; Group 3, negative control. The same four birds from each group were sampled at 0, 7, 10, 14, and 28 dpi. Each of these birds in a group is shown by a unique symbol (circle, diamond, star, or triangle) and has different color for each dpi. By following each symbol and color, the microbial composition of that particular bird at different sampling points can be evaluated. In addition, blood from birds killed at 7, 10, 14, and 28 dpi (without any symbols) were also analyzed.

**FIGURE 9:**
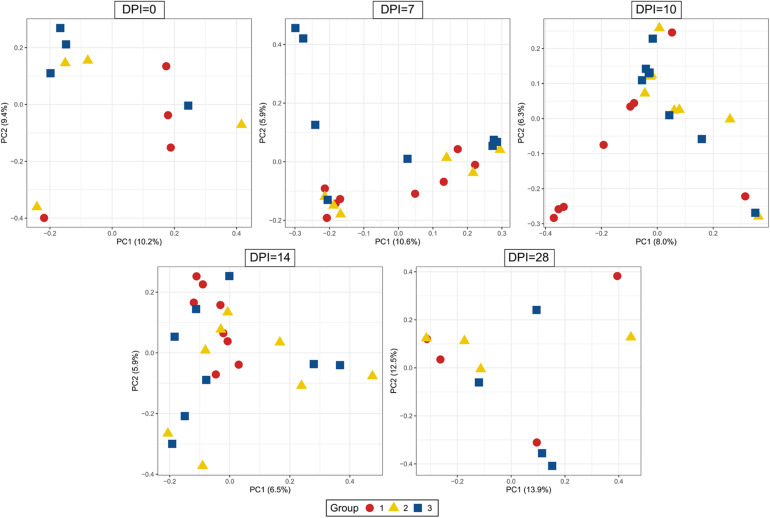
Principal Coordinate Analysis (PCoA) of beta diversity using Bray-Curtis distances. Each dot corresponds to one bird. Group 1, infected with *H. meleagridis* + *lux*-tagged *E. coli*; Group 2, infected with *lux*-tagged *E. coli*; Group 3, negative control. dpi denotes for days post *H. meleagridis* infection.

The evaluation of the microbial composition and diversity in the blood did not reveal any specific patterns related to groups or days of sampling in regards to alpha diversity measures (Figure 7) or relative proportion of most abundant genera (Figure 8). The relative proportion of the 20 most abundant genera did not reach 50% in most of the samples and many were dominated by taxa which were unable to be classified according to SILVA database, reflecting the complexity of the environment. Most of the 16S rRNA gene amplicon sequences from the blood were assigned to *Staphylococcus* (4.5% of total 16S rRNA gene amplicon sequences), *Streptococcus* (4.2%), *Corynebacterium* (3.9%), *Anaerococcus* (3.8%), *Veillonella* (3.4%), *Pseudomonas* (3.3%), *Actinomyces* (3.1%), *Haemophilus* (3.0%), and *Serratia* (2.8%). The microbial composition in the blood varied widely within the same or between different birds at all sampling time points (Figure 8). The evaluation of beta-diversity analysis using Bray Curtis distances also revealed a wide heterogeneity irrespective of groups and days of sampling (Figure 9).

### Relationship Between the Gut and Blood Microbiota

In order to evaluate the potential effect of *H. meleagridis* to translocate bacteria or their genetic elements from the cecum to the blood stream, common ASVs in these two environments were analyzed. The number of ASVs shared between the blood and the cecum of birds in groups 1, 2, and 3 were 85, 27, and 120, respectively. Most of the ASVs shared in the negative control birds (group 3) were assigned to *Lactobacillus, Bifidobacteruim, Rikenellaceae* RC9 gut group and members of *Ruminococcaceae and Lachnospiraceae*. In contrary, numbers of ASVs assigned to *Pseudomonas, Staphylococcus, Corynebacterium, Streptococcus, Helicobacter, Actinomyces, Pasteurella, Micrococcus* and *Serratia* genera were highest in birds in group 1 followed by group 2 (Table 2).

**TABLE 2:** List of 15 most important commensal and pathogenic bacterial genera and their ASVs shared between cecum and blood.

Genera	Number of ASVs		
	Group 1^a^	Group 2	Group 3
*Ruminococcaceae* members	12	10	49
*Lachnospiraceae* members	4	6	22
*Lactobacillus*	3	1	5
*Rikenellaceae RC9 gut group*	*3*	0	7
*Bifidobacterium*	0	0	2
*Escherichia*	2	2	2
*Serratia*	2	0	0
*Pseudomonads*	2	1	0
*Staphylococcus*	3	1	0
*Corynebacterium*	4	0	1
*Streptococcus*	2	0	0
*Helicobacter*	0	1	0
*Actinomyces*	1	0	0
*Pasteurella*	1	0	0
*Micrococcus*	3	0	0

*^*a*^Group 1, infected with *H. meleagridis* and *lux*-tagged *E. coli;* group 2, infected only with *lux*-tagged *E. coli;* group 3, negative control.*

### Immunohistochemistry

*Histomonas meleagridis* parasites were detected in all four caeca and one liver sample from group 1 (data not shown). All other samples were negative.

**FIGURE 10:**
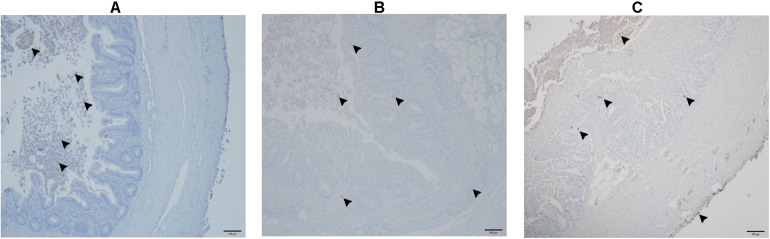
Detection of *E. coli* in the cecum with immunohistochemistry. *E. coli* are only present in the lumen of cecum (arrow head) in a negative control bird from group 3 **(A)**; presence of *E. coli* shown with arrowheads in the lumen, and mucosa of cecum in a co-infected bird in group 1 **(B)**; profound presence of *E. coli* indicated with arrowheads entire cecal wall in the dead bird which also showed severe colibacillosis **(C)**.

The number of *E. coli*-positive organs identified by IHC is presented in Table 3. *E. coli* was found in cecal lumen of all birds. In negative control birds, there was no infiltration of *E. coli* in the cecal wall (Figure 10A) or in systemic organs. In the co-infected group, the bacteria were observed in the cecal tissue (Figure 10B) in 7 birds with sporadic occurrence in liver (*n* = 2), spleen (*n* = 2) and heart (*n* = 1). In group 2, the dead bird, showed a profound amount of bacterial cells distributed throughout the cecal wall (Figure 10C), liver, spleen and heart. Other birds of the same group were positive in cecum (*n* = 4), liver (*n* = 2), spleen (*n* = 2), and heart (*n* = 1).

**TABLE 3:** Detection of *E. coli* with immunohistochemistry in organs.

Group^a^	Organs				
	Cecum		Liver	Spleen	Heart
	Lumen	Wall			
1 (*n* = 16)	16^b^	7	2	2	1
2 (*n* = 16)	16	4 + dead bird^c^	2 + dead bird	2 + dead bird	1 + dead bird
3 (*n* = 16)	16	0	0	0	0

*^*a*^Group 1, infected with *H. meleagridis* and *lux*-tagged *E. coli*; group 2, infected only with *lux*-tagged *E. coli*; group 3, negative control. ^*b*^Number of positive samples. ^*c*^Bird that died due to colibacillosis.*

### Isolation and Quantification of *lux*-Tagged *E. coli*

In direct plating, *lux*-tagged *E. coli* was re-isolated from lungs of one bird in the co-infected group (group 1) at 7, 10, 14, and 28 dpi, and from the ovary of one bird at 7 dpi. The dead bird from group 2 was positive for the bacterial strain in the lung, ovary and heart. Re-isolations of *lux*-tagged *E. coli* from blood samples of all birds at 7, 10, 14, and 28 dpi were negative.

**FIGURE 11:**
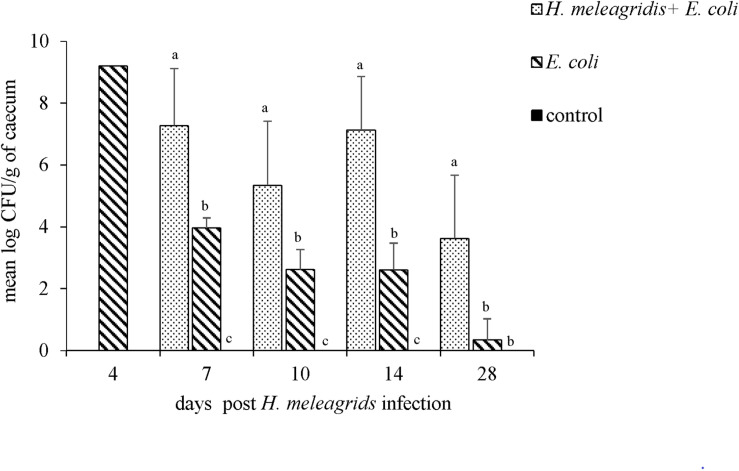
Quantification of the *lux*-tagged *E. coli* in the cecum of infected birds. One bird died from group 2 at 4 dpi. At 7, 10, 14, and 28 dpi sampling were performed in four birds from each group with an exception at 7 dpi when only three birds from group 2 were killed. Results are represented as means ± SEM. Numbers of bacteria are expressed as (log CFU/g). Bars with different letters within each sampling point denote significantly different values.

Upon bacterial quantification, at all sampling points, the average bacterial count of *lux*-tagged *E. coli* in the cecum was significantly higher in birds from group 1 than in group 2 (Figure 11). The bird that died at 4 dpi harbored 9.21 log CFU/g of *lux*-tagged *E. coli* in the cecum. In systemic organs, at 7 dpi, one bird from group 1 was positive in liver that contained 2.31 log CFU/g of the bacteria. At 14 dpi, one bird from group 2 contained 1.32 log CFU/g of lux-tagged *E. coli* in liver. The dead bird contained 9.17 and 8.16 log CFU/g of the bacteria in liver and spleen, respectively.

### Correlation of Macroscopic Cecal Lesion Score With Other Parameters

**FIGURE 12:**
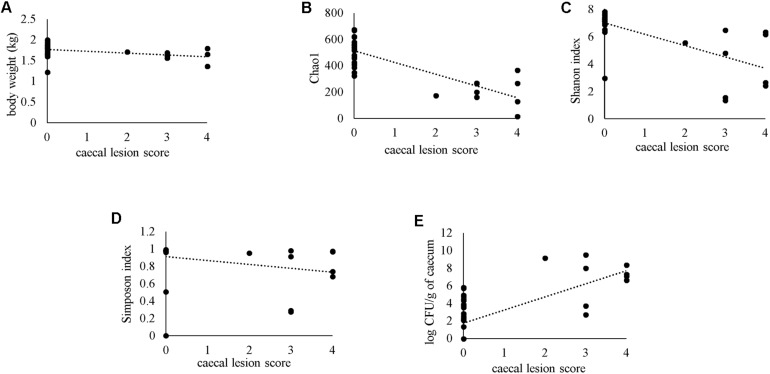
Spearman’s rank correlation of macroscopic cecal lesion score with body weight **(A)**, alpha diversity metrics in the caeca **(B–D)** or bacterial load of *lux*-tagged *E. coli* in the cecum **(E)**.

Severity of gross cecal LSs negatively correlated with body weight (*r* = −0.477) and species diversity indices in the cecum; Chao1 (*r* = −0.664), Shannon (*r* = −0.645) and Simpson (*r* = −0.548) (Figures 12A–D). A positive correlation of macroscopic LSs with the log CFU/g count of *lux*-tagged *E. coli* in the cecum was observed (*r* = 0.627; Figure 12E). All the above correlations were statistically significant.

## Discussion

The chicken intestine harbors a huge population of commensal microbes as well as pathogenic bacteria such as APEC. Infection of birds with *H. meleagridis* causes damage of the cecum and its mucosa. Thus the present study was aimed to investigate the influence of *H. meleagridis* on APEC-induced health parameters in layer chickens, especially in context of their translocation.

*H. meleagridis* cannot be grown *in vitro* without bacteria. In order to avoid the influence of the co-cultivated bacterial strain on any of the objectives of the study, *E. coli* DH5α was used which do not have potential to colonize tissues *in vivo* (Ganas et al., 2012). A large number of *E. coli* types are present in the chicken intestine that are genotypically indistinguishable (Ewers et al., 2005). Thus, it is impossible to differentiate the inoculated strain from native *E. coli* types in the gut which complicates experimental studies. In order to overcome this limitation, the avian pathogenic *E. coli* strain used for infecting the birds in the actual study was chromosomally tagged with the *lux*ABCDE operon which enabled visualization of the particular *E. coli* strain with bioluminescence. Along with a negative control group, two *E. coli*-infected groups, one with and the other without *H. meleagridis* were included in the actual study. The experimental design allowed to observe any effect of APEC infection compared to healthy birds and to further analyze if such effects are influenced by a co-infection with *H. meleagridis*. Thus, an additional group of birds infected only with *H. meleagridis* was not necessary to test the hypotheses of the actual study. Furthermore, it has to be kept in mind that no axenic culture of *H. meleagridis* is available due to growth characteristics of the parasite *in vitro*.

Birds less likely develop colibacillosis after oral infection with *E. coli* as compared to respiratory routes (Goren, 1978; Landman et al., 2013). However, oral and cloacal routes were preferred to deposit the bacteria in the gut and to monitor their dissemination. Interestingly, one bird infected only with *lux*-tagged *E. coli* developed colibacillosis. This fortifies the general understanding that the intestine can act as a reservoir and the residing pathogenic *E. coli* can develop the disease once a favorable opportunity arises within the host. Microscopic lesions noticed in systemic organs could be due to the translocation of bacteria or bacterial products from the gut (Truscott et al., 1974). In the actual study, lesions in the caeca due to *H. meleagridis* were seen as previously reported in experimentally or naturally infected adult chickens (Liebhart et al., 2013; Dolka et al., 2015). Such lesions were temporary, reaching the peak at 14 dpi. In a previous study in turkeys, it was demonstrated that the xenic culture of *H. meleagridis* can induce the disease approximately 1 week earlier than with monoxenic culture of the protozoal parasite co-cultivated with DH5α (Ganas et al., 2012). Similar to this, we also observed highest lesion scores in caeca only at 14 dpi, which is slightly later than it was reported earlier in chickens with xenic culture of *H. meleagridis* (Zahoor et al., 2011). Thus, it can be supposed that the time point when birds were inoculated with APEC was a bit too early leading to the hypothesis that the clinical pictures could have been different when APEC would have been administered after onset of cecal lesions. But there is no previously published information of this kind. Nevertheless, co-infection of birds with *E. coli* and *H. meleagridis* led to compromised health of birds as demonstrated by a reduced body weight and increased severity of systemic lesions.

The cecum is a complex ecosystem and various factors including infections influence the composition of highly variable microbiomes (Diaz Carrasco et al., 2019). In this study, it was observed that *lux*-tagged *E. coli* alone did not cause a pronounced effect on the cecal microbiota, which is likely to be correlated with the absence of macroscopic and microscopic lesions. In contrast, co-infection with *H. meleagridis* led to prominent shift in the population structure with more pronounced effect seen in caeca with higher lesions scores. The correlation between microbial richness and diversity indices with cecal lesion scores suggested that the severe pathology induced by *H. meleagridis* was responsible for the greater misbalance observed in cecal mucosa-associated microbiota. Infection of chickens with other pathogens such as *Clostridium perfringens*, *Campylobacter jejuni*, and *Eimeria tenella* are also known to have similar effects on the gut microbiota (Awad et al., 2016; Lin et al., 2017; Chen et al., 2020). We also observed that co-infection in birds was associated with a reduction in abundance of *Lactobacillus, Faecalibacterium* and some members of *Ruminococcaceae*, which are highly beneficial for the host metabolism (Rychlik, 2020). The lack of deeper taxonomic assignation of *Ruminococcaceae* bacteria can be explained by the lack of representation of certain animal gastrointestinal tract microbiota in 16S rRNA gene databases (Stewart et al., 2019). Probiotics with *Lactobacillus* are shown to have anti-parasitic properties and to improve body weight gain and feed conversion rate (Bozkurt et al., 2014). Likewise, bacteria of the *Ruminococcaceae* family are commonly involved in the metabolism of complex carbohydrates (Blake et al., 2015) and their lower abundance was shown to cause a decrease in body weight after *Eimeria tenella* infection in chickens (Chen et al., 2020). Bacteria such as *Escherichia*, *Helicobacter* and *Fusobacterium*, that were usually increased in co-infected birds in group 1 are known to have zoonotic importance and negative effects on the host (Pan and Yu, 2014; Pandit et al., 2018). The data from the present study showed that co-infection of chickens with *H. meleagridis* and *E. coli* severely disturbs the composition of resident microbiota, allowing dominance of potentially pathogenic microorganisms and suppression of the beneficial commensal ones, ultimately affecting the health status of birds.

So far, data from the microbiota analysis in the blood of chickens are very scarce. In fact, only a single study was reported which showed that the most abundant bacterial genera in broilers were *Cloacibacterium, Methylobacterium, Dechloromonas, Propionibacterium, Staphylococcus, Corynebacterium, Pseudomonas, Campylobacter, Streptococcus*, an*d Bacillus* (Mandal et al., 2016). These observations are not completely in agreement with the data from the actual study. Different age, genetics and environment of birds influence on the microbiota (Kers et al., 2018), thus the discrepancy between studies is not unexpected. Independent of this, bacteria might use blood as a vehicle to reach to the systemic organs, which could be the reason that we observed less stable microbial profiles in the blood as compared to the ones in cecum. Bacterial overgrowth, immunodeficiency and intestinal mucosal damage are major factors for the bacterial translocation from the gut to the blood (Berg, 1995). The finding of some ASVs shared between cecum and blood samples in all groups suggested possible microbial translocation, even in healthy birds. Translocation of microbes or microbial products to blood can occur through intact intestinal mucosa via dendritic cells (Niess et al., 2005) or goblet cells (McDole et al., 2012). The higher number of shared ASVs in the negative control birds were assigned to the group of commensal bacteria that are preferably found in the caecal mucous layer (Richards et al., 2019). Thus, sloughing of the mucus membrane caused by *H. meleagridis* is the most likely reason to observe less number of these bacteria in the blood of co-infected birds. Our data suggest that the cecal dysbiosis induced by *H. meleagridis* in chickens influences the microbial population in the blood as it was shown previously in mice (Runkel et al., 1991).

With IHC, it was possible to localize *E. coli* cells in tissues and identify a potential translocation pathway. In contrast to negative control birds, *E. coli* showed the tendency to colonize the cecal lumen in all infected birds. However, co-infection with *H. meleagridis* favored tissue penetration of *E. coli*, which is most likely due to the damaged cecal mucosal wall caused by the protozoal infection. In the dead bird, peritonitis along with profuse number of *E. coli* in all the subepithelial layers, including serosa of the cecum was observed. The observation provided a thought-provoking evidence that intestinal *E. coli* get access to the peritoneum after successful penetration of the cecal wall, which might lead to colibacillosis in chickens. This hypothesis is further supported by the finding that *E. coli* was mainly detected on the serosal surface of systemic organs, which presumably came from the peritoneum.

The frequency of re-isolation of the *lux*-tagged *E. coli* strain from the systemic organs was not very high. This is similar to a previous study that showed only few *E. coli*-positive livers and lungs but an extensive cecal colonization of *E. coli* following an oral inoculation in axenic turkeys (Dominick and Jensen, 1984). The higher colonization of caeca by pathogenic *E. coli* in co-infected birds might be the consequence of dysbiosis leading to a lower proportion of commensal bacteria or due to the direct interaction of parasite-bacteria, which was demonstrated *in vitro* (Ganas et al., 2012). Higher number and less clearance of pathogenic *E. coli* from the gut could increase the chances of cecal tissue penetration by the bacteria. Secondly, aerosolization of the bacteria from the contaminated feces might be more efficient due to increased bacterial shedding, as indicated by the higher rate of *lux*-tagged *E. coli* re-isolation from lungs in co-infected birds. Microbiota analysis in systemic organs of chickens is not very common, however, recent data suggested the existence of microbial interaction between intestine and respiratory system (Ngunjiri et al., 2019). In future studies, it would be worth to further investigate the gut-lung axis in chickens infected with *E. coli*.

## Conclusion

Laying chickens co-infected with a *lux*-tagged avian pathogenic *E. coli* and *H. meleagridis* showed higher microscopic lesions in systemic organs, reduced body weight and severe dysbiosis with consequences on the reduction of commensal bacteria. Penetration of cecal wall by the intestinal *E. coli* was supported by the infection with *H. meleagridis*, suggesting a plausible mechanism for the induction of colibacillosis in chickens affected with histomonosis. Higher numbers of pathogenic *E. coli* colonizing in cecal tissues in the presence of *H. meleagridis* increase prospects of colibacillosis in chickens when a favorable opportunity arise in the host. Altogether, the data presented here provided novel insights on the interaction between *H. meleagridis* and the microbiota, especially *E. coli* in chickens.

## Data Availability Statement

Raw sequence reads were uploaded to the NCBI BioProject databank (PRJNA647676).

## Ethics Statement

The animal study was reviewed and approved by the Institutional Ethics Committee and the National Authority according to §8ff of the law for Animal Experiments, Tierversuchsgesetz-TVG (License Number GZ.68.205/0220-V/3b/2018).

## Author Contributions

CH, MH, and SP conceived and designed the study. MA, CH, DL, and SP performed the animal trial and sampling. MA and MD prepared the samples for illumina sequencing. NMQ, MD, and ES carried out microbiota analyses. SP worked on bioluminescent tagging and its validation with technical inputs from IB. MA and DL performed and analyzed the histology and immunohistochemistry. TH prepared the *H. meleagridis* culture. MA and SP drafted the manuscript. All authors reviewed the manuscript and provided their inputs.

## Conflict of Interest

The authors declare that the research was conducted in the absence of any commercial or financial relationships that could be construed as a potential conflict of interest.

## Footnotes

1 https://www.viennabiocenter.org

2 http://www.bioinformatics.babraham.ac.uk/projects/fastqc/

3 https://docs.qiime2.org/2019.7/plugins/available/feature-table/

4 https://www.r-project.org

5 https://docs.qiime2.org/2019.7/plugins/available/diversity/

6 https://docs.qiime2.org/2019.7/plugins/available/taxa/

7 https://cran.r-project.org/web/packages/dplyr/

8 https://cran.r-project.org/web/packages/lemon/index.html

## Supplementary Material

Supplementary Figure 1 Bioluminescent colonies of *lux*-tagged PA14/17480/5-ovary visualized under IVIS.

Supplementary Figure 2 Body weight (kg) of commercial layers at different time points after infection with *H. meleagridis* + *lux*-tagged *E. coli*, only with *lux*-tagged *E. coli* and negative control.

Supplementary Figure 3 Average bacterial counts measured by qPCR in cecum **(A)** and blood buffy coat **(B)** in each group at different time points. Significant difference is indicated with an asterisk.

Supplementary Table 1 Histopathology lesion scoring scheme.

Supplementary Table 2 Comparison of alpha diversity indices in caeca.

Supplementary Table 3 PERMANOVA analysis of beta diversity in caeca.
