# Supplementary figures and images for: Co-infection of Chicken Layers With Histomonas meleagridis and Avian Pathogenic Escherichia coli Is Associated With Dysbiosis, Cecal Colonization and Translocation of the Bacteria From the Gut Lumen
Source: Front Microbiol. 2020 Oct 30;11:586437. doi: 10.3389/fmicb.2020.586437 (PMC7661551; doi:10.3389/fmicb.2020.586437)

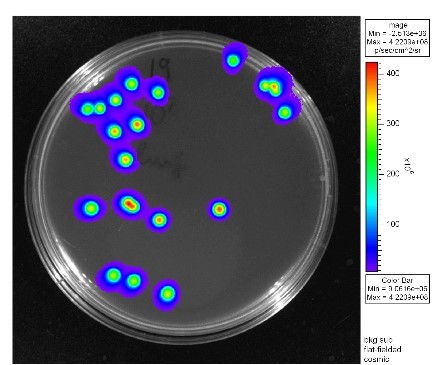

Supplement: Supplementary Figure 1 — Bioluminescent colonies of lux-tagged PA14/17480/5-ovary visualized under IVIS. [file Image_1.JPEG]

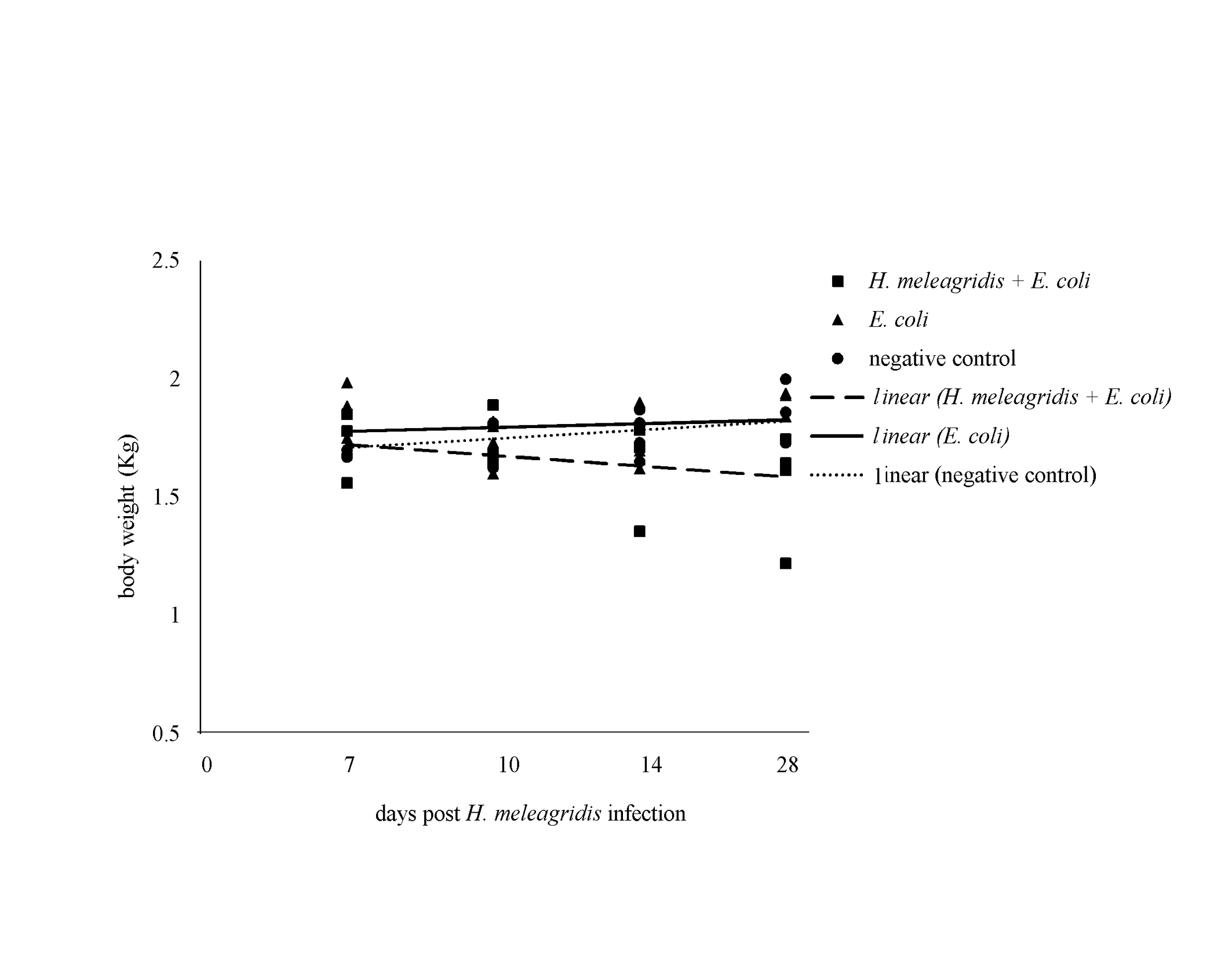

Supplement: Supplementary Figure 2 — Body weight (kg) of commercial layers at different time points after infection with H. meleagridis + lux-tagged E. coli, only with lux-tagged E. coli and negative control. [file Image_2.TIF]

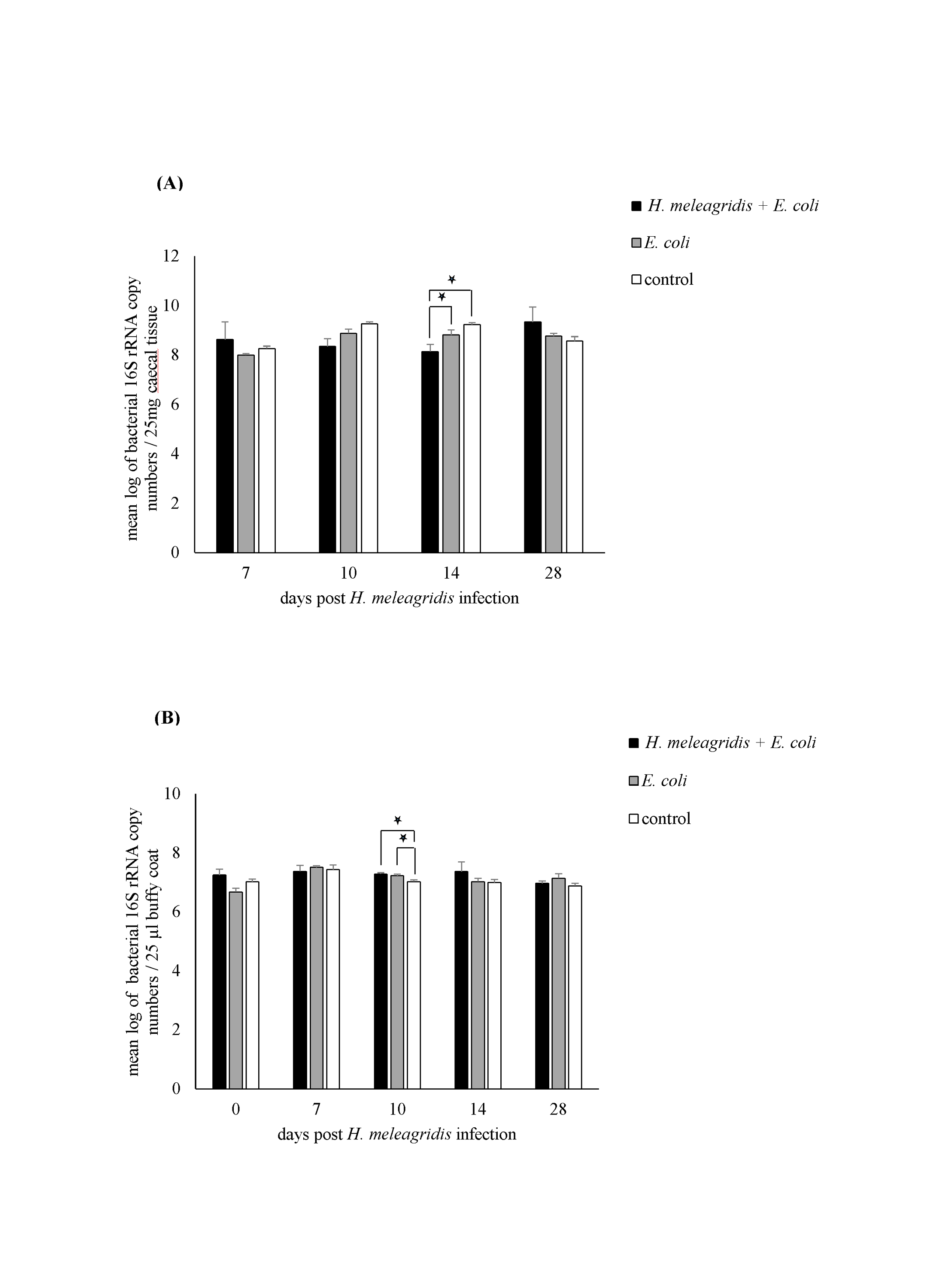

Supplement: Supplementary Figure 3 — Average bacterial counts measured by qPCR in cecum (A) and blood buffy coat (B) in each group at different time points. Significant difference is indicated with an asterisk. [file Image_3.TIF]
