# Supplementary material for: Co-infection of Chicken Layers With Histomonas meleagridis and Avian Pathogenic Escherichia coli Is Associated With Dysbiosis, Cecal Colonization and Translocation of the Bacteria From the Gut Lumen
Source: Front Microbiol. 2020 Oct 30;11:586437. doi: 10.3389/fmicb.2020.586437 (PMC7661551; doi:10.3389/fmicb.2020.586437)
Supplement: Supplementary Table 1 — Histopathology lesion scoring scheme. [file Table_1.DOCX]

**Supplementary Table 1.** Histopathology lesion scoring scheme

| **score** | **caecum** | **liver** | **heart** | **spleen** |
| --- | --- | --- | --- | --- |
| 0 | normal | normal | normal | normal |
| 1 | epithelial erosion with infiltration of inflammatory cells that exceed into submucosa | multifocal hepatocellular necrosis and infiltration of inflammatory cells | focal infiltration of inflammatory cells into endocardium, myocardium and/or epicardium | minor to moderate infiltration of heterophils in splenic tissue |
| 2 | extensive mucosal damage with infiltration of inflammatory cells throughout all caecal wall layers | coalescent hepatocellular necrosis and infiltration of inflammatory cells | multifocal infiltration of inflammatory cells into endocardium, myocardium and/or epicardium | severe heterophilic influx and /or lymphoid depletion in splenic tissue |
