# Supplementary material for: Co-infection of Chicken Layers With Histomonas meleagridis and Avian Pathogenic Escherichia coli Is Associated With Dysbiosis, Cecal Colonization and Translocation of the Bacteria From the Gut Lumen
Source: Front Microbiol. 2020 Oct 30;11:586437. doi: 10.3389/fmicb.2020.586437 (PMC7661551; doi:10.3389/fmicb.2020.586437)
Supplement: Supplementary Table 2 — Comparison of alpha diversity indices in caeca. [file Table_2.DOCX]

**Supplementary Table 2: Comparison of alpha diversity indices in caeca**

| **group** | **7 dpi^a^** | | | **10 dpi** | | | **14 dpi** | | | **28 dpi** | | |
| --- | --- | --- | --- | --- | --- | --- | --- | --- | --- | --- | --- | --- |
|  | **Chao1** | **Shannon** | **Simpson** | **Chao1** | **Shannon** | **Simpson** | **Chao1** | **Shannon** | **Simpson** | **Chao1** | **Shannon** | **Simpson** |
| *H. meleagridis*  *+ E. coli* | 360.5 | 4.34 | 0.63^a^ | 381.6 | 5.51 | 0.90 | 249.3^a^ | 5.20^a^ | 0.9^a^ | 377.4 | 6.58 | 0.97 |
| *E. coli* | 472.2 | 7.09 | 0.98^ab^ | 539.6 | 7.09 | 0.98 | 511.8^ab^ | 7.37^ab^ | 0.99^b^ | 582.6 | 7.49 | 0.99 |
| control | 585.7 | 7.48 | 0.99^b^ | 578 | 7.48 | 0.97 | 578^b^ | 7.59^b^ | 0.99^b^ | 592 | 7.29 | 0.98 |
| *P* value | 0.302 | 0.114 | 0.027 | 0.793 | 0.491 | 0.584 | 0.018 | 0.023 | 0.039 | 0.173 | 0.059 | 0.058 |

^a^ dpi: days post *H. meleagridis* infection. Values are expressed as mean of birds in a group at each sampling event. Values with different superscripts in a column differ significantly
