# Supplementary material for: Co-infection of Chicken Layers With Histomonas meleagridis and Avian Pathogenic Escherichia coli Is Associated With Dysbiosis, Cecal Colonization and Translocation of the Bacteria From the Gut Lumen
Source: Front Microbiol. 2020 Oct 30;11:586437. doi: 10.3389/fmicb.2020.586437 (PMC7661551; doi:10.3389/fmicb.2020.586437)
Supplement: Supplementary Table 3 — PERMANOVA analysis of beta diversity in caeca. [file Table_3.DOCX]

**Supplementary Table 3: PERMANOVA analysis of beta diversity in caeca**

| **pseudo-F** | ***p*-value^c^** | **q-value** | **groups^b^** | **dpi^a^** |
| --- | --- | --- | --- | --- |
| 1.66293 | 0.214 | 0.286 | 1 vs 2 | 7 |
| 1.815483 | 0.286 | 0.286 | 1 vs 3 |  |
| 1.817545 | 0.028 | 0.084 | 2 vs 3 |  |
| 1.432479 | 0.029 | 0.032 | 1 vs 2 | 10 |
| 1.495209 | 0.032 | 0.032 | 1 vs 3 |  |
| 2.036034 | 0.028 | 0.032 | 2 vs 3 |  |
| 1.910139 | 0.032 | 0.048 | 1 vs 2 | 14 |
| 2.068712 | 0.028 | 0.048 | 1 vs 3 |  |
| 1.1783 | 0.117 | 0.117 | 2 vs 3 |  |
| 1.351913 | 0.034 | 0.034 | 1 vs 2 | 28 |
| 1.598374 | 0.03 | 0.034 | 1 vs 3 |  |
| 2.850487 | 0.034 | 0.034 | 2 vs 3 |  |

^a^ dpi: days post *H. meleagridis* infection; ^b^ group 1: infected with *H. meleagridis* and *lux*-tagged *E. coli*, group 2: infected with *lux*-tagged *E. coli*, group 3: negative control; ^c^ *p* ≤ 0.05 was considered as statistically significant
